# Cost-Efficient Distributed Learning via Combinatorial Multi-Armed Bandits †

**DOI:** 10.3390/e27050541

**Published:** 2025-05-20

**Authors:** Maximilian Egger, Rawad Bitar, Antonia Wachter-Zeh, Deniz Gündüz

**Affiliations:** 1 School of Computation, Information and Technology, Technical University of Munich, 80333 Munich, Germany; rawad.bitar@tum.de (R.B.); antonia.wachter-zeh@tum.de (A.W.-Z.); 2 Department of Electrical and Electronic Engineering, Imperial College London, London SW7 2AZ, UK; d.gunduz@imperial.ac.uk

**Keywords:** distributed machine learning, multi-armed bandits, stochastic gradient descent, straggler mitigation

## Abstract

We consider the distributed stochastic gradient descent problem, where a main node distributes gradient calculations among *n* workers. By assigning tasks to all workers and waiting only for the *k* fastest ones, the main node can trade off the algorithm’s error with its runtime by gradually increasing *k* as the algorithm evolves. However, this strategy, referred to as *adaptive k-sync*, neglects the cost of unused computations and of communicating models to workers that reveal a straggling behavior. We propose a cost-efficient scheme that assigns tasks only to *k* workers, and gradually increases *k*. To learn which workers are the fastest while assigning gradient calculations, we introduce the use of a combinatorial multi-armed bandit model. Assuming workers have exponentially distributed response times with different means, we provide both empirical and theoretical guarantees on the regret of our strategy, i.e., the extra time spent learning the mean response times of the workers. Furthermore, we propose and analyze a strategy that is applicable to a large class of response time distributions. Compared to adaptive *k*-sync, our scheme achieves significantly lower errors with the same computational efforts and less downlink communication while being inferior in terms of speed.

## 1. Introduction

We consider a distributed machine learning setting, in which a central entity, referred to as the *main node*, possesses a large amount of data on which it wants to run a machine learning algorithm. To speed up the computations, the main node distributes the computation tasks to several *worker* machines. The workers compute smaller tasks in parallel and send back their results to the main node, which then aggregates the partial results to obtain the desired result of the large computation. A naive distribution of the tasks to the workers suffers from the presence of *stragglers*, that is, slow or unresponsive workers [1,2].

The negative effect of stragglers can be mitigated by assigning redundant computations to the workers and ignoring the responses of the slowest ones [3,4]. However, in gradient descent algorithms, assigning redundant tasks to the workers can be avoided when a (good) estimate of the gradient loss function is sufficient. On a high level, gradient descent is an iterative algorithm requiring the main node to compute the gradient of a loss function at every iteration based on the current model. Simply ignoring the stragglers is equivalent to stochastic gradient descent (SGD) [5,6], which advocates computing an estimate of the gradient of the loss function at every iteration [2,7]. As a result, SGD trades off the time spent per iteration with the total number of iterations for convergence, or until a desired result is reached.

The authors of [8] show that for distributed SGD algorithms, it is faster for the main node to assign tasks to all the workers but wait for only a small subset to return their results. In the strategy proposed in [8], called *adaptive k-sync*, in order to improve the convergence speed, the main node increases the number of workers it waits for as the algorithm evolves in iterations. Despite reducing the runtime of the algorithm, i.e., the total time needed to reach the desired result, this strategy requires the main node to transmit the current model to all available workers and pay for all computational resources while only using the computations of the fastest ones. This concern is particularly relevant in scenarios where computational resources are rented from external providers, making unused or inefficiently utilized resources financially costly [9]. It is equally important when training on resource-constrained edge devices, where communication and computation capabilities are severely limited [10,11].

In this work, we take into account the cost of employing workers and transferring the current model to the workers. In contrast to [8], we propose a communication- and computation-efficient scheme that distributes tasks only to the fastest workers and waits for the completion of all their computations. However, in practice, the main node does not know in advance which workers are the fastest. For this purpose, we introduce the use of a stochastic multi-armed bandit (MAB) framework to learn the speed of the workers while efficiently assigning them computational tasks.

Stochastic MABs, introduced in [12], are iterative algorithms designed to maximize the gain of a user gambling with multiple slot machines, termed “armed bandits”. At each iteration, the user is allowed to pull one arm from the available set of armed bandits. Each arm pull yields a random reward following a known distribution with an unknown mean. The user wants to design a strategy to learn the expected reward of the arms while maximizing the accumulated rewards. Stochastic combinatorial MABs (CMABs) were introduced in [13] and model the behavior when a user seeks to find a combination of arms that reveals the best overall expected reward.

Following the literature on distributed computing [3,14], we model the response times of the workers by independent and exponentially distributed random variables. We additionally assume that the workers are heterogeneous, i.e., have different mean response times. To apply MABs to distributed computing, we model the rewards by the response times. Our goal here is to use MABs to minimize the expected response time; hence, we would like to minimize the average reward, instead of maximizing it. Under this model, we show that compared to adaptive *k*-sync, using an MAB to learn the mean response times of the workers on the fly cuts the average cost (reflected by the total number of ‘worker employments’) but comes at the expense of significantly increasing the total runtime of the algorithm.

### 1.1. Related Work

#### 1.1.1. Distributed Gradient Descent

Assigning redundant tasks to the workers and running distributed gradient descent is known as gradient coding [4,9,15,16,17,18]. Approximate gradient coding is introduced to reduce the required redundancy and run SGD in the presence of stragglers [19,20,21,22,23,24,25]. The schemes in [17,18] use redundancy but no coding to avoid encoding/decoding overheads. However, assigning redundant computations to the workers increases the computation time spent per worker and may slow down the overall computation process. Thus, Refs. [2,7,8] advocate for running distributed SGD without redundant task assignment to the workers.

In [7], the convergence speed of the algorithm was analyzed in terms of the wall-clock time rather than the number of iterations. It was assumed that the main node waits for *k* out of *n* workers and ignores the rest. Gradually increasing *k*, i.e., gradually decreasing the number of tolerated stragglers as the algorithm evolves, is shown to increase the convergence speed of the algorithm [8]. In this work, we consider a similar analysis to the one in [8]; however, instead of assigning tasks to all the workers and ignoring the stragglers, the main node only employs (assigns tasks to) the required number of workers. To learn the speed of the workers and choose the fastest ones, we use ideas from the literature on MABs.

#### 1.1.2. MABs

Since their introduction in [12], MABs have been extensively studied for decision-making under uncertainty. An MAB strategy is evaluated by its *regret*, defined as the difference between the actual cumulative reward and the one that could be achieved should the user know the expected reward of the arms a priori. Refs. [26,27] introduced the use of upper confidence bounds (UCBs) based on previous rewards to decide which arm to pull at each iteration. Those schemes are said to be asymptotically optimal since the increase of their regret becomes negligible as the number of iterations goes to infinity.

In [28], the regret of a UCB algorithm is bounded for a finite number of iterations. Subsequent research aims to improve on this by introducing variants of UCBs, e.g., KL-UCB [29,30], which is based on Kullback–Leibler (KL)-divergence. While most of the works assume a finite support for the reward, MABs with unbounded rewards were studied in [29,30,31,32], where in the latter, the variance factor is assumed to be known. In the class of CMABs, the user is allowed to pull multiple arms with different characteristics at each iteration. The authors of [13] extended the asymptotically efficient allocation rules of [26] to a CMAB scenario. General frameworks for the CMAB with bounded reward functions are investigated in [33,34,35,36]. The analysis in [37,38] for linear reward functions with finite support is an extension of the classical UCB strategy and is most closely related to our work.

### 1.2. Contributions and Outline

Our main contribution is the design of a computation-efficient and communication-efficient distributed learning algorithm through the use of the MAB framework. We apply MAB algorithms from the literature and adapt them to our distributed computing setting. We show that the resulting distributed learning algorithm outperforms state-of-the-art algorithms in terms of computation and communication costs. The costs are measured in terms of the number of ‘worker employments’, whether the results of the corresponding computations carried out by the workers are used by the main node or not. On the other hand, the proposed algorithm requires a longer runtime due to learning the speed of the workers. Parts of the results in this paper were previously presented in [39].

The rest of the paper is organized as follows. After a description of the system model in Section 2, in Section 3, we introduce a round-based CMAB model based on lower confidence bounds (LCBs) to reduce the cost of distributed gradient descent. Our cost-efficient policy increases the number of employed workers as the algorithm evolves. In Section 4, we introduce and theoretically analyze an LCB that is particularly suited to exponential distributions and requires low computational complexity at the main node. To improve the performance of our CMAB, we investigate in Section 5 an LCB that is based on KL-divergence, and generalizes to all bounded reward distributions and those belonging to the canonical exponential family. This comes at the expense of a higher computational complexity for the main node. In Section 6, we provide simulation results for linear regression to underline our theoretical findings. Section 8 concludes the paper.

## 2. System Model and Preliminaries

**Notations.** Vectors and matrices are denoted in bold lower and upper case letters, e.g., z and Z, respectively. For integers κ, τ with κ<τ, the set $\left\{\kappa ,\kappa +1,\ldots ,\tau \right\}$ is denoted by [κ,τ], and $\left[\tau \right]≔\left\{1,\ldots ,\tau \right\}$. Sub-gamma distributions are expressed by shape α and rate β, i.e., $\mathrm{Sub}\Gamma \left(\alpha ,\beta \right)$, and sub-Gaussian distributions by variance $\sigma^{2}$, i.e., $\mathrm{SubG}\left(\sigma^{2}\right)$. The identity function 1{z} is 1 if *z* is true, and 0 otherwise. Throughout the paper, we use the terms arm and worker interchangeably.

We denote by $X\in R^{m\times d}$ a data matrix with *m* samples, where each sample $x_{\ell }\in R^{d}$, $\ell \in \left[1,m\right]$, corresponds to the *ℓ*-th row of X. Let $y\in R^{m}$ be the vector containing the labels $y_{\ell }$ for every sample $x_{\ell }$. The goal is to find a model $w\in R^{d}$ that minimizes an additively separable loss function $F\left(X,y,w\right)≔\sum_{\ell =1}^{m}F\left(x_{\ell },y_{\ell },w\right)$, i.e., to find $w_{★}=\arg\min_{w\in R^{d}}F\left(X,y,w\right)$.

We consider a distributed learning model, where a main node possesses the large dataset (X,y), and a set of *n* workers available for outsourcing the computations needed to run the minimization. We assume that the dataset (X,y) is stored on a shared memory, and can be accessed by all *n* workers. The main node employs a distributed variant of the iterative stochastic gradient descent (SGD) algorithm. At each iteration *j*, the main node employs some number of workers, indexed by A(j), to run the necessary computations. More precisely, each worker i∈A(j) will perform the following actions: (i) The worker receives the model $w_{j}\in R^{d}$ from the main node; (ii) it samples a random subset (batch) $X_{i,j}\in R^{s\times d}$ of X, and $y_{i,j}\in R^{s}$ of y consisting of $s=\frac{m}{b}$ samples (for ease of analysis, we assume that *b* divides *m*, which can be satisfied by adding all-zero rows to X and corresponding zero labels to y). The size of the subset is constant and fixed throughout the training process; (iii) worker i∈A(j) computes a *partial gradient* estimate $\nabla F(X_{i,j},y_{i,j},w_{j})$ based on $X_{i,j}$ and $y_{i,j}$, as well as the model $w_{j}$, and returns the gradient estimate to the main node. The main node waits for R(j)⊆A(j) responsive workers and updates the model w as follows:

(1) $$\begin{array}{cc} w_{j+1} & =w_{j}-\frac{\eta }{|R(j)|\;\cdot \;s}\sum_{i\in R(j)}\nabla F\left(X_{i,j},y_{i,j},w_{j}\right)\\ \; & =w_{j}-\frac{\eta }{\left|R(j)\right|\;\cdot \;s}\sum_{i\in R(j)}\sum_{\ell \in V_{i,j}}\nabla F\left(x_{\ell },y_{\ell },w\right), \end{array}$$

where η denotes the learning rate, and by $V_{i,j}$ we denote the set of indices of all samples in $X_{i,j}$. According to [7,40], fixing the value of $\left|R(j)\right|=k$ and running *j* iterations of gradient descent with a mini-batch size of s·k results in an expected deviation from the optimal loss $F^{★}$, bounded as follows. This result holds under the standard assumptions detailed in [7,40], i.e., a Lipschitz-continuous gradient with bounds on the first and second moments of the objective function, characterized by *L* and $\sigma^{2}$, respectively, strong convexity with parameter *c*, the stochastic gradient being an unbiased estimate, and a sufficiently small learning rate η:

(2) $$\begin{array}{cc} \;\;E\left(k,j\right) & =E\left[F\left(k,w_{j}\right)-F^{★}\right]\leq \;\;\underset{\mathrm{error}\;\mathrm{floor}}{\underset{︸}{\frac{\eta L\sigma^{2}}{2cks}}}\;\;+\underset{\mathrm{transient}\;\mathrm{behavior}}{\underset{︸}{{\left(1-\eta c\right)}^{j}\left(F\left(w_{0}\right)-F^{★}-\frac{\eta L\sigma^{2}}{2cks}\right)}}. \end{array}$$

As the number of iterations goes to infinity, the influence of the transient behavior vanishes, and what remains is the contribution of the error floor.

As shown in [7], algorithms that fix *k* throughout the process exhibit a trade-off between the time spent per iteration, the final error achieved by the algorithm, and the number of iterations required to reach that error. The authors show that a careful choice of *k* can reduce the total runtime. However, *k* need not be fixed throughout the process. Hence, in [8,41,42], the authors study a family of algorithms that change the value of *k* as the algorithm progresses in the number of iterations, further reducing the total runtime. The main drawback of all those works is that they employ *n* workers at each iteration and only use the computation results of k≤n of them. Thus, resulting in a waste of worker employment. In this work, we tackle the same distributed learning problem, but with a budget constraint *B*, measured by the total number of worker employments. Algorithms of [7,8,41,42] exhaust the budget in B/n iterations, as they employ *n* workers per iteration. On the contrary, we achieve a larger number of iterations by employing the necessary number *k* of workers at each iteration and using all their results. We impose the constraint of k≤b for a certain parameter b≤n, i.e., we restrict the number of workers that can be employed in parallel. The main challenge is to choose the fastest *k* workers at all iterations to reduce the runtime of the algorithm.

## 3. CMAB for Distributed Learning

We focus on the interplay between communication/computation costs and the runtime of the algorithm. For a given desired value of the loss to be achieved (cf. (2), parametrized by a value k=b), we study the runtime of algorithms constrained by the total number of workers employed. The number of workers employed serves as a proxy for the total communication and computation costs incurred. Our design choices (in terms of increasing *k*) stem from the optimization provided in [8], where the runtime was optimized as a function of *k* while neglecting the associated costs. We use a combinatorial multi-armed bandit framework to learn the response times of the workers while using them to run the machine learning algorithm.

We group the iterations into *b* rounds, such that at iterations within round $r\in \left[1,b\right]$, the main node employs $\left|A(j)\right|=r$ workers and waits for all of them to respond, i.e., A(j)=R(j). As in [8], we let each round *r* run for a predetermined number of iterations. The number of iterations per round is chosen based on the underlying machine learning algorithm to optimize the convergence behavior.More precisely, at a switching iteration $j=T_{r}$, the algorithm advances to round r+1. Algorithms for convergence diagnostics can be used to determine the best possible switching times. For example, the authors of [8,42] used Pflug’s diagnostics [43] to measure the state of convergence by comparing the statistics of consecutive gradients. Improved measures were studied in [44] and can be directly applied to this setting. Furthermore, established early stopping tools apply; however, studying them is not the focus of this work. We define $T_{0}≔0$, i.e., the algorithm starts in round one, and $T_{b}$ is the last iteration, i.e., the algorithm ends in round *b*. The total budget *B* is defined as $B≔\sum_{r=1}^{b}r\;\cdot \;\left(T_{r}-T_{r-1}\right)$, which gives the total number of worker employments. We use the number of worker employments as a measure of the algorithm’s efficiency, as it directly impacts both computation and communication costs. The goal of this work is to reach the best possible performance with the fewest employments while reducing the total runtime. Since the number of iterations per round is chosen based on the underlying machine learning algorithm, *best arm identification* techniques, such as those studied in [45], are not adequate for this setting. Identifying the best arm in a certain round may require a larger number of iterations, thus delaying the algorithm.

Following the literature on distributed computing [3,9,14,46], we assume exponentially distributed response times of the workers; that is, the response time $Z_{i}^{j}$ of worker *i* in iteration *j*, resulting from the sum of communication and computation delays, follows an exponential distribution with rate $\lambda_{i}$ and mean $\mu_{i}=\frac{1}{\lambda_{i}}$, i.e., $Z_{i}^{j}∼\exp\left(\lambda_{i}\right)$. The minimum rate of all the workers is $\lambda_{\min}≔\min_{i\in \left[1,n\right]}\lambda_{i}$. The goal is to assign tasks only to the *r* fastest workers. The assumption of exponentially distributed response times is motivated by its wide use in the literature of distributed computing and storage. However, we note that the theoretical guarantees we will provide hold, with slight modifications, for a much larger class of distributions that exhibit the properties of sub-Gaussian distributions on the left tail, and those of sub-gamma distributions on the right tail. This holds for many heavy-tailed distributions, e.g., the Weibull or the log-normal distribution for certain parameters.

We denote by policy π a decision process that chooses the *r* expected fastest workers. The optimal policy $\pi^{★}$ assumes knowledge of the $\mu_{i}$ and chooses *r* workers with the smallest $\mu_{i}$. Rather than fixing a certain value of *r* throughout the training process, it can be shown that starting with small values of *r* and gradually increasing the number of concurrently employed workers is beneficial for the algorithm’s convergence, with respect to both time and efficiency, as measured by the number of worker employments. Hence, we choose this policy as a baseline. However, in practice, the $\mu_{i}$s are unknown in the beginning. Thus, our objective is twofold. First, we want to find confident estimates ${\hat{\mu }}_{i}$ of the mean response times $\mu_{i}$ to correctly identify (explore) the fastest workers, and second, we want to leverage (exploit) this knowledge to employ the fastest workers as much as possible, rather than investing in unresponsive/straggling workers.

To trade off this exploration–exploitation dilemma, we utilize the MAB framework, where each arm i∈[1,n] corresponds to a different worker, and *r* arms are pulled at each iteration. A *superarm*$A^{r}\left(j\right)\subseteq \left[n\right]$ with $\left|A^{r}\left(j\right)\right|=r$ is the set of indices of the arms pulled at iteration *j*, and $A^{r,★}$ is the optimal choice containing the indices of the *r* workers with the smallest means.

For all workers, indexed with *i*, we maintain a counter $T_{i}\left(j\right)$ for the number of times this worker has been employed until iteration *j*, i.e., $T_{i}\left(j\right)=\sum_{y=1}^{j}1\left\{i\in A^{r}\left(y\right)\right\}$, and a counter $M_{i}\left(j\right)$ for the sum of its response times, i.e., $M_{i}\left(j\right)=\sum_{y=1}^{j}1\left\{i\in A^{r}\left(y\right)\right\}Z_{i}^{y}$. The LCB of a worker is a measure based on the empirical mean ${\hat{\mu }}_{i}\left(j\right)=\frac{M_{i}\left(j\right)}{T_{i}\left(j\right)}$ and the number of samples $T_{i}\left(j\right)$ chosen such that the true mean $\mu_{i}$ is unlikely to be smaller. As the number of samples grows, the LCB of worker *i* approaches ${\hat{\mu }}_{i}$. A policy π is then a rule to compute and update the LCBs of the *n* workers, such that at iteration $j\in [T_{r-1}+1,T_{r}]$, the *r* workers with the smallest LCBs are pulled. The choice of the confidence bounds significantly affects the performance of the model and will be analyzed in Section 4 and Section 5. A summary of the CMAB policy and the steps executed by the workers is given in Algorithm 1.
**Algorithm 1** Combinatorial multi-armed bandit policy**Require:** Number of workers *n*, budget b≤n  1:**Initialize:** ∀i∈[n]: ${\mathrm{LCB}}_{i}\left(0\right)\leftarrow -\infty$ and $T_{i}\left(0\right)\leftarrow 0$  2:**for** r=1,…,b **do**    ▹ Run (combinatorial) MAB with *n* arms pulling *r* at a time   3:    **for** $j=T_{r-1}\;+\;1,\ldots ,T_{r}$ **do**  4:        $\forall i\in \left[n\right]:$ calculate ${\mathrm{LCB}}_{i}\left(j-1\right)$  5:        Choose $A^{r}\left(j\right)$, i.e., *r* workers with the minimum ${\mathrm{LCB}}_{i}\left(j-1\right)$ where $i\in \left[n\right]$  6:        Every worker $i\in A^{r}\left(j\right)$ computes a gradient estimate $\nabla F(X_{i,j},y_{i,j},w_{j})$ and sends it to the main node  7:        $\forall i\in A^{r}\left(j\right)$: Observe response time $Z_{i}^{j}$  8:        $\forall i\in A^{r}\left(j\right)$: Update statistics, i.e., $T_{i}\left(j\right)=T_{i}\left(j-1\right)+1$, ${\hat{\mu }}_{i}\left(j\right)=\frac{Z_{i}^{j}+T_{i}\left(j-1\right)\;\cdot \;{\hat{\mu }}_{i}\left(j-1\right)}{T_{i}\left(j\right)}$  9:        Update model $w_{j}$ according to (1)10:    **end for**11:**end for**

**Remark 1.**  *Instead of using a CMAB policy, one could consider the following non-combinatorial strategy. At every round, one arm is declared to be the best and is removed from the MAB policy in future rounds. That is, at round r, r−1 arms are pulled deterministically, and an MAB policy is used on the remaining n−r+1 arms to determine the next best arm. While this strategy simplifies the analysis, it could result in a linear increase in the regret. The number of iterations per round is decided based on the performance of the machine learning algorithm. This number can be small and may not be sufficient to determine the best arm with high probability. Thus, making the non-combinatorial MAB more likely to commit to sub-optimal arms.*

In contrast to most works on MABs, we minimize an unbounded objective, i.e., the overall computation time $Z_{A^{r}\left(j\right)}^{j}≔\max_{i\in A^{r}\left(j\right)}Z_{i}^{j}$ at iteration *j*. This corresponds to waiting for the slowest worker. The expected response time of a superarm $A^{r}\left(j\right)$ is then defined as $\mu_{A^{r}\left(j\right)}≔E\left[Z_{A^{r}\left(j\right)}^{j}\right]$ and can be calculated according to Proposition 1.

**Proposition 1.**  *The mean of the maximum of independently distributed exponential random variables with different means, indexed by a set I, i.e., $Z_{p}∼\exp\left(\lambda_{p}\right)$, p∈I, is given as follows:*

(3) $$\begin{array}{c} E\left[\max_{p\in I}Z_{y}\right]=\sum_{S\in P\left(I\right)\setminus \emptyset }{\left(-1\right)}^{|S|-1}\frac{1}{\sum_{\xi \in S}\lambda_{\xi }}, \end{array}$$

*with $P\left(I\right)$ denoting the power set of I.*

**Proof.**  The proof is given in Section 7.1. □

**Proposition 2.**  *The variance of the maximum of independently distributed exponential random variables with different means, indexed by a set I, i.e., $Z_{p}∼\exp\left(\lambda_{p}\right)$, p∈I, is given as follows:*

$$\begin{array}{c} Var\left[\max_{p\in I}Z_{i}\right]=\sum_{S\in P\left(I\right)\setminus \emptyset }{\left(-1\right)}^{|S|-1}\;\left(2{\left(\frac{1}{\sum_{\xi \in S}\lambda_{\xi }}\right)}^{2}-\frac{1}{\sum_{\xi \in S}\lambda_{\xi }}\right) \end{array}$$

*with $P\left(I\right)$ denoting the power set of I.*

**Proof.**  The proof follows similar lines to that of Proposition 1 and is omitted for brevity. □

The suboptimality gap of a chosen (super-)arm describes the expected difference in time compared to the optimal choice.

**Definition 1.**  *For a superarm $A^{r}\left(j\right)$, and for $A_{\mathrm{worst}}^{r}$, defined as the set of indices of the r slowest workers, we define the following superarm suboptimality gaps:*

(4) $$\begin{array}{cc} \Delta_{A^{r}\left(j\right)} & ≔\mu_{A^{r}\left(j\right)}-\mu_{A^{r,★}},\\ \Delta_{A^{r},\max} & ≔\mu_{A_{\mathrm{worst}}^{r}}-\mu_{A^{r,★}}. \end{array}$$

*For ν≤r, $A_{\nu }^{r}\left(j\right)$ and $A_{\nu }^{r,★}$, denote the indices of the $\nu^{\mathrm{th}}$ fastest worker in $A^{r}\left(j\right)$ and $A^{r,★}$, respectively. Then, we define the suboptimality gap for the *employed *arms as follows:*

$$\begin{array}{cc} \delta_{A_{\nu }^{r}\left(j\right)} & ≔\mu_{A_{\nu }^{r}\left(j\right)}-\mu_{A_{\nu }^{r,★}}. \end{array}$$

*Let $W^{r}$ denote the set of all superarms with cardinality r. We define the minimum suboptimality gap for *all *the arms as follows:*

(5) $$\begin{array}{cc} \delta_{\min} & ≔\min_{r\in \left[b\right],A^{r}\in W^{r}}\;\;\min_{\nu \in \left[r\right]:\mu_{A_{\nu }^{r}}>\mu_{A_{\nu }^{r,★}}}\delta_{A_{\nu }^{r}}. \end{array}$$

**Example 1.**  *For mean worker computation times given by $U=\left\{\mu_{1},\ldots ,\mu_{n}\right\}$, we obtain the following: $\delta_{\min}\geq \min_{\kappa ,\tau \in U:\kappa >\tau }\kappa -\tau .$*

**Definition 2.**  *We define the regret $R_{j}^{\pi }$ of a policy π run until iteration j as the expected difference in the runtime of the policy π compared to the optimal policy $\pi^{★}$, i.e.,*

$$\begin{array}{cc} R_{j}^{\pi } & =\sum_{r\in \left[b\right]:j>T_{r-1}}E\left[\sum_{y=T_{r-1}+1}^{T_{r}}Z_{A^{r}\left(y\right)}^{y}\right]-\sum_{r\in \left[b\right]:j>T_{r-1}}\left(\min\left\{j,T_{r}\right\}-T_{r-1}\right)\;\;\min_{A^{r}\in W^{r}}\;\mu_{A^{r}}. \end{array}$$

Definition 2 quantifies the overhead in total time spent by π to learn the average speeds of the workers and will be analyzed in Section 4 and Section 5 for two different policies, i.e., choices of LCBs. In Theorem 1, we provide a runtime guarantee of an algorithm using a CMAB for distributed learning as a function of the regret $R_{j}^{\pi }$ and the number of iterations *j*.

**Theorem 1.**  *Given a desired ϵ>0, the time until policy π reaches iteration j is bounded from above as follows:*

$$t_{j}^{\pi }\;\leq \;R_{j}^{\pi }+\sum_{r=1}^{b}\;1\left\{j>T_{r-1}\right\}\mu_{A^{r,★}}\left(\min\left\{j,T_{r}\right\}-T_{r-1}\right)\;\left(1+\epsilon \right)$$

*with probability*

$$\mathrm{Pr}\left(j\right)\geq \prod_{r\in \left[b\right]:j>T_{r-1}}\left(1-\frac{\sigma_{A^{r,★}}^{2}}{\mu_{A^{r,★}}^{2}\left(\min\left\{j,T_{r}\right\}-T_{r-1}\right)\epsilon^{2}}\right).$$

*The mean $\mu_{A^{r,★}}$ and variance $\sigma_{A^{r,★}}^{2}$ can be calculated according to Propositions 1 and 2.*

**Proof.**  The proof is given in Section 7.2. □

The theorem bounds the runtime required by a policy π to reach a certain iteration *j* as a function of the time it takes an optimal policy to reach iteration *j* (determined by the response time of the optimal arms at each round *r*), and the regret of the policy π that is executed. The regret quantifies the gap to the optimal policy. To give a complete performance analysis, in Proposition 3, we provide a handle on the expected deviation from the optimal loss as a function of the number of iterations *j*. Combining the results of Theorem 1 and Proposition 3, we obtain a measure on the expected deviation from the optimal loss with respect to time. Proposition 3 is a consequence of the convergence characteristics of the underlying machine learning algorithm, showing the expected deviation from the optimal loss reached at a certain iteration *j*.

**Proposition 3.**  *The expected deviation from the optimal loss at iteration j in round r of an algorithm using CMAB for distributed learning can be bounded by $E\left(k,j^{\prime }\right)$ as in (2) for k=r and $j^{\prime }=j_{r,\mathrm{begin}}+j-T_{r-1}$, where $j_{1,\mathrm{begin}}=0$, and for $r\in \left[2,b\right]$,*

$$\begin{array}{cc} j_{r,\mathrm{begin}}= & \frac{\log\left(\frac{\eta L\sigma^{2}}{2cs}\left(\frac{1}{r-1}-\frac{1}{r}\right)+\alpha^{j_{r-1,\mathrm{end}}}\left(E_{0}-\frac{\eta L\sigma^{2}}{2c(r-1)s}\right)\right)}{\log\left(\alpha \right)}-\frac{\log\left(E_{0}-\frac{\eta L\sigma^{2}}{2crs}\right)}{\log\left(\alpha \right)}, \end{array}$$

*with $j_{r,\mathrm{end}}≔j_{r,\mathrm{begin}}+T_{r}-T_{r-1}$, $E_{0}≔F\left(w_{0}\right)-F^{★}$ and α≔1−ηc.*

**Proof.**  The statement holds because, at each round *r*, the algorithm follows the convergence behavior of an algorithm with a mini-batch of fixed size rs. For algorithms with a fixed mini-batch size, we only need the number of iterations run to bound the expected deviation from the optimal loss. However, in round r>1 and iteration *j*, the algorithm has advanced differently than with a constant mini-batch of size rs. Thus, we need to recursively compute the equivalent number of iterations $j^{\prime }\in \left[T_{r-1}+1,T_{r}\right]$ that have to be run for a fixed mini-batch of size rs to finally apply (2).

Therefore, we have to compute $j_{r,\mathrm{begin}}$, which denotes the iteration for a fixed batch size rs with the same error as for a batch size of (r−1)s at the end of the previous round r−1, denoted by $j_{r-1,\mathrm{end}}≔j_{r-1,\mathrm{begin}}+T_{r-1}-T_{r-2}$. To calculate $j_{r,\mathrm{begin}}$, the following condition must hold: $E\left(r,j_{r,\mathrm{begin}}\right)=E\left(r-1,j_{r-1,\mathrm{end}}\right)$. This can be repeated recursively, until we can use $j_{1,\mathrm{end}}$. For round r=1, the problem is trivial. Alternatively, one could also use the derivation in [40], Equation (4.15), and recursively bound the expected deviation from the optimal loss in round *r* based on the expected deviation at the end of the previous round r−1, i.e, use $E[F\left(w_{T_{r-1}}\right)-F^{★}]$ instead of $E_{0}$. □

In this section, the LCBs are treated as a black box. In Section 4 and Section 5, we present two different LCB policies along with their respective performance guarantees.

**Remark 2.**  *The explained policies can be seen as an SGD algorithm, which gradually increases the mini-batch size. In the machine learning literature, this is one of the approaches considered to optimize convergence. Alternatively, one could also use b workers with a larger learning rate from the start and gradually decrease the learning rate to trade off the error floor in (2) against runtime.*

*For the variable learning rate approach, one can use a slightly adapted version of our policies, where $\left|A^{r}\left(j\right)\right|=b$ is fixed. If the goal is to reach a particular error floor, our simulations show that the latter approach achieves this target faster than the former. This, however, only holds under the assumption that the chosen learning rate in (1) is sufficiently small, i.e., the scaled learning rate at the beginning of the algorithm still leads to convergence.*

*However, if one seeks to optimize the convergence speed at the expense of reaching a slightly higher error floor, simulations show that decaying the learning rate is slower because the learning rate is limited to ensure convergence.*

*Optimally, one would combine both approaches by starting with the maximum possible learning rate, gradually increasing the number of workers per iteration until reaching b, and then decreasing the learning rate to reach the best error floor.*

## 4. Confidence Radius-Based Policy

Motivated by [28], we present a confidence radius-based policy $\pi_{\mathrm{cr}}$ that is computationally light for the main node. With this policy, in iteration $j\in [T_{r-1}+1,T_{r}]$ the superarm $A^{r}\left(j\right)$ is chosen as the *r* arms with the lowest LCBs calculated as follows:

$${\mathrm{LCB}}_{i}\left(j-1\right)≔\left\{\begin{array}{cc} -\infty & \mathrm{if}\;T_{i}\left(j-1\right)=0\\ {\hat{\mu }}_{i}\left(j-1\right)-\theta_{i}\left(j-1\right) & \mathrm{otherwise}, \end{array}\right.$$

where ${\hat{\mu }}_{i}\left(j\right)\frac{M_{i}\left(j\right)}{T_{i}\left(j\right)}$. The choice of the confidence radius $\theta_{i}\left(j\right)$ affects the performance of the policy and is based on the underlying reward distribution, chosen as $\theta_{i}\left(j\right)≔\sqrt{\frac{4f(j)}{T_{i}\left(j\right)}}+\frac{2f(j)}{T_{i}\left(j\right)}$, with f(j)=2log(j). By this particular choice, we can prove a bounded regret in the setting of minimizing outcomes subject to an exponential distribution. The estimates ${\hat{\mu }}_{i}\left(j\right)$ and the confidence radii are updated after every iteration according to the responses of the chosen workers. We give a performance guarantee for this confidence bound choice in terms of the regret in Theorem 2.

**Theorem 2.**  *The regret of the CMAB policy $\pi_{\mathrm{cr}}$, which uses the gradually increasing superarm size and selects arms based on LCBs with radius $\theta_{i}\left(j\right)≔\sqrt{\frac{4f(j)}{T_{i}\left(j\right)}}+\frac{2f(j)}{T_{i}\left(j\right)}$, where f(j)=2log(j), and under the assumption $\lambda_{\min}\geq 1$, is bounded above as follows (note that the assumption $\lambda_{\min}\geq 1$ is needed for our proof to hold. In practice, this assumption amounts to choosing the time unit of our theoretical model such that the average response time of each worker is less than one time unit):*

(6) $$\begin{array}{cc} R_{j}^{\pi_{\mathrm{cr}}}\leq & \max_{r\in \left[b\right]:j>T_{r-1}}\Delta_{A^{r},\max}\;\cdot \;n\;\cdot \;\left(\frac{48\log(j)}{\min\{\delta_{\min}^{2},\delta_{\min}\}}+1+u\;\cdot \;\frac{\pi^{2}}{3}\right), \end{array}$$

*where $u≔\max_{r\in \left[b\right]:j>T_{r-1}}r$.*

**Proof.**  The proof is given in Section 7.4. □

Proposition 3 shows that the regret at iteration *j* depends on the maximum suboptimality gap of a superarm over all rounds *r* executed up until the current iteration *j* as a measure of the worst case regret, and depends on the minimum suboptimality gaps as a measure of the difficulty of the exploration problem. The regret further increases logarithmically in the number of iterations *j*, which is a desirable property and reflects a successful exploration strategy that does not continuously commit to suboptimal arms in the long run. The regret bound in Theorem 2 is loose due to the use of $\Delta_{A^{r},\max}$ and $\delta_{\min}$ in Equation (6). Nevertheless, the bound reflects the same qualitative round-based behavior of the proposed CMAB policies (cf., Section 6). Choosing other parameters to tighten the bound leads to a cumbersome equation.

## 5. KL-Based Policy

The authors of [29] proposed using a KL-divergence-based confidence bound for MABs to improve the regret compared to classical UCB-based algorithms. Due to the use of KL-divergence, this scheme is applicable to reward distributions that have bounded support or belong to the canonical exponential family. Motivated by this, we extend this model to a CMAB for distributed machine learning, and define a policy $\pi_{\mathrm{kl}}$ that calculates LCBs according to the following:

(7) $$\begin{array}{c} \;\;\;{\mathrm{LCB}}_{i}\left(j\right)≔\min\left\{q<{\hat{\mu }}_{i}:T_{i}\left(j\right)\;\cdot \;D_{\mathrm{KL}}\left(p_{{\hat{\mu }}_{i}}∥p_{q}\right)\leq f\left(j\right)\right\},\; \end{array}$$

where $f\left(j\right)=\log\left(j\right)+3\log\left(\log\left(j\right)\right)$. This confidence bound, i.e., the minimum value for *q*, can be calculated using the Newton procedure for root finding by solving $e\left(j,{\hat{\mu }}_{i},q\right)≔T_{i}\left(j\right)\;\cdot \;D_{\mathrm{KL}}\left(p_{{\hat{\mu }}_{i}}∥p_{q}\right)-\log\left(j\right)=0$, and is thus computationally heavy for the main node. For exponential distributions with probability density function *p* parametrized by means ${\hat{\mu }}_{i}$ and *q*, respectively, the KL-divergence is given by $D_{\mathrm{KL}}\left(p_{{\hat{\mu }}_{i}}∥p_{q}\right)={\hat{\mu }}_{i}/q-\log\left({\hat{\mu }}_{i}/q\right)-1$. Its derivative can be calculated as $\partial D_{\mathrm{KL}}\left(p_{{\hat{\mu }}_{i}}∥p_{q}\right)/\partial q=1/q-{\hat{\mu }}_{i}/q^{2}$. With this at hand, the $\xi^{\mathrm{th}}$ Newton update is denoted as follows:

$$q_{\xi }=q_{\xi -1}-\frac{D_{\mathrm{KL}}\left(p_{{\hat{\mu }}_{i}}∥p_{q_{\xi -1}}\right)}{\partial D_{\mathrm{KL}}\left(p_{{\hat{\mu }}_{i}}∥p_{q_{\xi -1}}\right)/\partial q_{\xi -1}}$$

Note that $q_{0}$ must not be equal to ${\hat{\mu }}_{i}$. In case $q_{0}={\hat{\mu }}_{i}$, the first update step would be undefined since $\partial D_{\mathrm{KL}}\left(p_{{\hat{\mu }}_{i}}∥p_{q_{0}}\right)/\partial q_{0}$ would be 0. In addition, $q_{0}$ should be chosen smaller than ${\hat{\mu }}_{i}$, e.g., $q_{0}=0.01\;\cdot \;{\hat{\mu }}_{i}$. For this policy $\pi_{\mathrm{kl}}$, we give the worst-case regret in Theorem 3. To ease the notation, we write $D_{\mathrm{KL}}\left(\kappa ∥\tau \right)≔D_{\mathrm{KL}}\left(p_{\kappa }∥p_{\tau }\right)$.

**Theorem 3.**  *Let the response times of the workers be sampled from a finitely supported distribution or a distribution belonging to the canonical exponential family. Then, the regret of the CMAB policy $\pi_{\mathrm{kl}}$, with the gradually increasing superarm size and arms chosen based on a KL-based confidence bound ${\mathrm{LCB}}_{i}\left(j\right)≔\min\left\{q<{\hat{\mu }}_{i}:T_{i}\left(j\right)\;\cdot \;D_{\mathrm{KL}}\left(p_{{\hat{\mu }}_{i}}∥p_{q}\right)\leq f\left(j\right)\right\}$ with $f\left(j\right)=\log\left(j\right)+3\log\left(\log\left(j\right)\right)$, j>3, and $u=\;\;\;\;\max_{r\in \left[b\right]:j>T_{r-1}}\;\;\;\;r$ can be upper-bounded as follows:*

(8) Rjπ≤maxr∈uΔAr,max·n·u·exp−DKLϵ,min1+ϵDKL,maxf(j)−11−exp(−DKL,min/(1+ϵ))7log(log(j))+1+ϵDKL,minf(j)+exp−DKLϵ,min1+ϵDKL,maxf(j)−11−exp(−DKLϵ,min),

*where ϵ is a parameter that can be freely chosen, and we have the following:*

$$\begin{array}{cc} D_{\mathrm{KL},\max} & =\;\;\;\;\;\max_{r\in \left[b\right],U\in W^{r},\nu \in \left[r\right]:\mu_{U_{\nu }}>\mu_{A_{\nu }^{r,★}}}\;\;\;\;\;\;\;\;D_{\mathrm{KL}}\left(\mu_{U_{\nu }}∥\mu_{A_{\nu }^{r,★}}\right),\\ D_{\mathrm{KL},\min} & =\;\;\;\;\;\min_{r\in \left[b\right],U\in W^{r},\nu \in \left[r\right]:\mu_{U_{\nu }}>\mu_{A_{\nu }^{r,★}}}\;\;\;\;\;\;\;\;D_{\mathrm{KL}}\left(\mu_{U_{\nu }}∥\mu_{A_{\nu }^{r,★}}\right),\\ D_{\mathrm{KL}\epsilon ,\min} & =\;\;\;\;\;\min_{r\in \left[b\right],U\in W^{r},\nu \in \left[r\right]:\mu_{U_{\nu }}>\mu_{A_{\nu }^{r,★}}}\;\;\;\;\;\;\;\;D_{\mathrm{KL}}\left(\phi \left(\epsilon ,\mu_{U_{\nu }},\mu_{A_{\nu }^{r,★}}\right)∥\mu_{U_{\nu }}\right), \end{array}$$

*with $\mu_{A_{\nu }^{r,★}}<\phi \left(\epsilon ,\mu_{A_{\nu }^{r,★}},\mu_{U_{\nu }}\right)<\mu_{U_{\nu }}$ such that $D_{\mathrm{KL}}\left(\phi \left(\epsilon ,\mu_{A_{\nu }^{r,★}},\mu_{U_{\nu }}\right)∥\mu_{A_{\nu }^{r,★}}\right)=\frac{D_{\mathrm{KL}}\left(\mu_{U_{\nu }}∥\mu_{A_{\nu }^{r,★}}\right)}{\left(1+\epsilon \right)}$.*

**Proof.**  The proof is given in Section 7.5. □

Similar to Theorem 2, the dominating factors of the regret in Theorem 3 depend logarithmically on the number of iterations and linearly on the worst-case suboptimality gap across all superarms up until the round *r* that corresponds to iteration *j*. The KL terms reflect the difficulty of the problem of identifying the best possible superarm, dominated by the superarm that is closest to the optimal choice.

**Remark 3.**  *The main goal of Theorems 2 and 3 is to show that the expected computation time of such algorithms is bounded, and to study the qualitative behavior of the round-based exploration strategy of the proposed algorithms. The performance for practical applications is expected to be significantly better. Results from [47,48,49] could be used to prove possibly tighter regret bounds, which will be left for future work.*

**Remark 4.**  *Regret lower bounds for non-combinatorial stochastic bandits were established in [26], and later extended to linear and contextual bandits in [50,51], respectively. In the combinatorial setting, lower bounds under general reward functions were studied in [52], revealing the added complexity introduced by combinatorial action spaces. In non-combinatorial problems, the KL-UCB algorithm of [29] is known to match the lower bound of [26] in specific cases. While our algorithm builds on a variant of KL-UCB, the round-dependent structure of our combinatorial bandit setting introduces additional challenges for proving optimality guarantees. Deriving regret lower bounds in such settings remains an open problem.*

## 6. Numerical Simulations

### 6.1. Setting

Similar to [8], we consider n=50 workers with exponentially distributed response times whose means are chosen uniformly at random from $\left\{0.1,0.2,\cdots ,0.9\right\}$ such that $\lambda_{\min}\geq 1$. We limit the budget to b=20 parallel computations. We create m=2000 samples $x_{\ell }$ with d=100 entries, each drawn uniformly at random from [1, 10] with labels $y_{\ell }∼N\left(x_{\ell }^{T}w^{\prime },1\right)$, for some $w^{\prime }$ drawn uniformly at random from ${\left[1,\;100\right]}^{d}$. The model w is initialized uniformly at random as $w_{0}\in {\left[1,100\right]}^{d}$ and optimized subject to the least squares loss function $F\left(X,y,w\right)≔\frac{1}{2}{∥Xw-y∥}_{2}^{2}$ with learning rate $\eta =1\times 10^{-4}$. We assess the performance of the model by the error function $∥X^{+}{y-w∥}_{2}$, where $X^{+}$ denotes the pseudo-inverse of X, which quantifies the gap to the analytical solution, so that the analysis is largely data-independent and problem-independent. For all the simulations, we present the results averaged over at least ten rounds.

### 6.2. Switching Points

The switching points $T_{r}$, $r\in \left[b\right]$, are the iterations in which we advance from round *r* to r+1. In [8], Pflug’s method [43] is used to determine the $T_{r}$’s on the fly. However, this method is very sensitive to the learning rate [44,53], and may result in different $T_{r}$’s across different runs. While implicit model updates [53] or alternative criteria [44] can avoid this effect, we fix the switching points to ensure comparability across simulation runs. We empirically determine $T_{1}$ and the necessary statistics to calculate $T_{r}$ for $r\in \left[2,b\right]$ using (2).

### 6.3. Simulation Results for Confidence Radius Policy $\pi_{\mathrm{cr}}$

**Figure 1:**
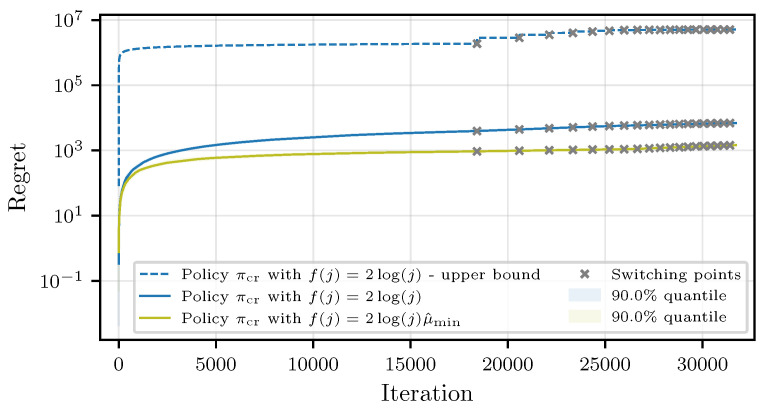
Comparison of the theoretical and simulated regret for $\pi_{\mathrm{cr}}$.

We first study the CMAB policy $\pi_{\mathrm{cr}}$, which was introduced in Section 4. Note that the confidence radii are not dependent on the actual mean worker response times. In case the workers have small response times, the confidence radii $\theta_{i}\left(j\right)$ might be very dominant compared to the empirical mean estimates ${\hat{\mu }}_{i}$, leading to a frequent employment of suboptimal workers. For practical purposes, it may, thus, be beneficial to use an adapted confidence radius with $f\left(j\right)=2\log\left(j\right){\hat{\mu }}_{\min}$, where ${\hat{\mu }}_{\min}=\min_{i\in \left[1,n\right]}{\hat{\mu }}_{i}$, which balances the confidence radius and the mean estimate. In Figure 1, we compare the theoretical regret guarantee in Theorem 2 to practical results for both confidence bound choices. As the theoretical guarantee is a worst-case analysis, the true performance is underestimated significantly. We can see that using $f\left(j\right)=2\log\left(j\right){\hat{\mu }}_{\min}$ significantly improves the regret. However, this comes at the cost of delaying the determination of the fastest workers. While with f(j)=2log(j), the policy correctly determines all *b* fastest workers in ten simulation runs, using $f\left(j\right)=2\log\left(j\right){\hat{\mu }}_{\min}$ in one out of ten simulations, the algorithm commits to a worker with a suboptimality gap of 0.1. This reflects the trade-off between the competing objectives of best arm identification and regret minimization discussed in [54]. However, since the fastest workers were eventually determined with an accuracy of 99.5%, the proposed adapted confidence bound seems to be a good choice in practice. Although the theoretical bound is rather loose and deviates from the simulations by up to some multiplicative factors, it shows the round-based behavior in a worst-case scenario.

**Figure 2:**
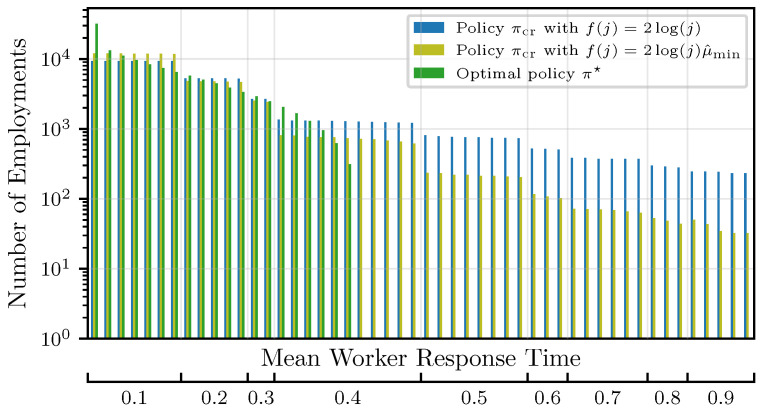
Number of worker employments for $\pi_{\mathrm{cr}}$ compared to the optimal policy $\pi^{★}$.

The number of worker employments is shown in Figure 2, with the workers sorted from the fastest to the slowest. As expected, compared to the optimal strategy, the LCB-based algorithms have to explore all workers, including suboptimal ones. With the adapted confidence bound and compared to f(j)=2log(j), suboptimal workers are employed significantly less due to the reason above. This also explains the different regrets in Figure 1.

### 6.4. Simulation Results for KL-Based Policy $\pi_{\mathrm{kl}}$

**Figure 3:**
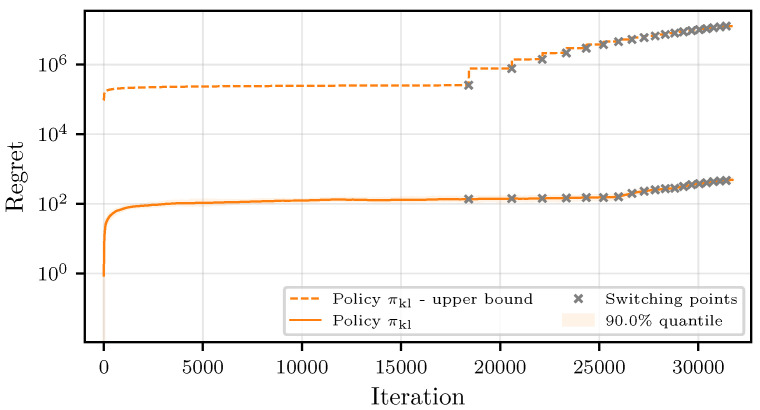
Comparison of the theoretical and simulated regret for $\pi_{\mathrm{kl}}$.

**Figure 4:**
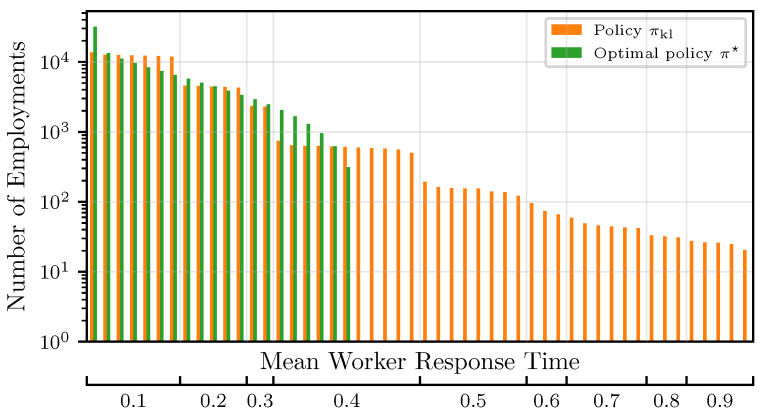
Number of worker employments for $\pi_{\mathrm{kl}}$ compared to the optimal policy $\pi^{★}$.

The strategy proposed in Section 5 introduces additional computational overhead for the main node to run a numerical procedure for calculating the LCBs due to the missing analytical closed-form solution. Depending on the computational resources of the main node, this overhead might outweigh the benefits of using the KL-based policy. However, the convergence rate improves significantly compared to the strategy in Section 4. This is reflected by the regret in Figure 3, where we find the regret bound again as a very pessimistic overestimate. The best workers in this case were determined with an accuracy of 99.0%, showing that the impacts of misclassification can almost be neglected and the regret improves by a factor of approximately ten compared to the results in Section 6.3. The improvement in regret follows directly from the reduced amount of suboptimal worker employments, which is depicted in Figure 4.

**Remark 5.**  *One could try to find confidence bounds that further minimize the cumulative regret. However, as shown in [54], the goals of regret minimization and best-arm identification become contradictory at some point. That is, one can either optimize an algorithm toward very confidently determining the best arms out of all available ones, or one could seek to optimize the cumulative regret to the maximum extent. Since in this case we are concerned with both objectives, the goal was to find a policy satisfying them simultaneously.*

### 6.5. Comparison to Adaptive k-Sync

**Figure 5:**
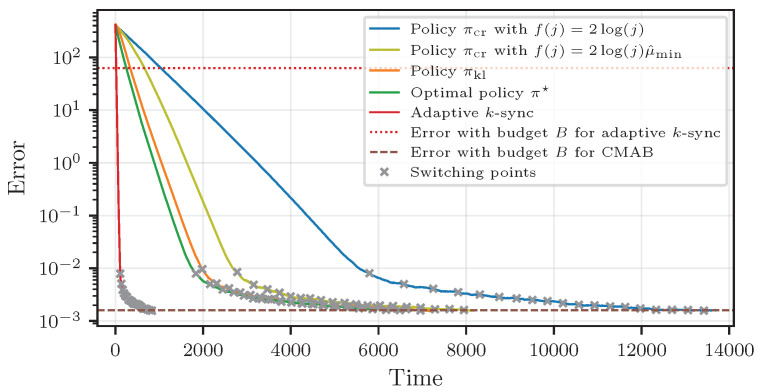
Comparison to adaptive *k*-sync [8] with limited budget *B*.

In Figure 5, we analyze the convergence of the algorithms from Section 4 (with two different f(j)) and Section 5, and compare with the optimal policy $\pi^{★}$ and the adaptive *k*-sync scheme from [8]. We note that this analysis is given for completeness. Studying the merits of adaptive algorithms was the goal of many works, e.g., [8,41,42], and our methodology directly applies to such techniques, providing efficiency in the number of worker employments, thereby improving the computation and communication efficiency at the expense of a slower runtime.

**Speed.** As waiting for the *r* fastest out of n>r workers is, on average, faster than waiting for all *r* workers, the adaptive *k*-sync strategy from [8] is significantly faster than our proposed scheme. Comparing $\pi^{★}$ to the performance of $\pi_{\mathrm{cr}}$ with f(j)=2log(j), learning the mean worker speeds slows down the convergence by a factor of almost three. This is because the chosen confidence radius mostly dominates the mean response time estimates of the workers, leading to an emphasis on exploration, i.e., more confident estimates at the expense of sampling slow workers more often. While the confidence bound adapted by ${\hat{\mu }}_{\min}$ accounts for this drawback, $\pi_{\mathrm{kl}}$ achieves the best results. However, this comes at the expense of more computational load to calculate the LCBs.

**Worker employment.** Considering the same cost, our proposed scheme is able to achieve significantly better results. In particular, with a total budget of $B<1.3\times 10^{5}$ computations, the CMAB-based strategy reaches an error of $\approx 2\times 10^{-3}$ while adaptive *k*-sync achieves an error of only $\approx 6\times 10^{1}$.

**Communication.** The CMAB schemes had to transfer in total $B<1.3\times 10^{5}$ models to the workers, while adaptive *k*-sync occupied the transmission link from the main node to the workers $n\;\cdot \;T_{b}>1.5\times 10^{6}$ times. Thus, the downlink communication cost is reduced by more than a factor of ten, whereas the load on the uplink channel from the workers to the main node is $B<1.3\times 10^{5}$ for both strategies. Consequently, the total amount of channel occupations has been reduced by more than 80%, i.e., 2B instead of $B+n\;\cdot \;T_{b}$.

## 7. Proofs

### 7.1. Proof of Proposition 1

Let $F_{Z}$ be the cumulative distribution function of random variable Z, and let $P\left(I\right)$ be the power set of I. Consider exponentially distributed random variables indexed by a set I, i.e., $Z_{p}∼\exp\left(\lambda_{p}\right)$, p∈I, with different rates $\lambda_{p}$ and the cumulative distribution function $F_{Z_{p}}\left(x\right)=1-e^{-\lambda_{p}x}$. Then, we can derive the maximum order statistics, i.e., the expected value of the largest of their realizations, as follows:

$$\begin{array}{cc} E\left[\max_{p\in I}Z_{p}\right] & =\int_{0}^{\infty }\left(1-F_{\max_{p\in I}Z_{p}}\left(x\right)\right)dx\\ \; & =\int_{0}^{\infty }\left(1-\prod_{p\in I}F_{Z_{p}}\left(x\right)\right)dx\\ \; & =\int_{0}^{\infty }\left(1-\prod_{p\in I}\left(1-e^{-\lambda_{p}x}\right)\right)dx\\ \; & =\int_{0}^{\infty }\left(1-\sum_{S\in P\left(I\right)}{\left(-1\right)}^{\left|S\right|}e^{-\sum_{\xi \in S}\lambda_{\xi }x}dx\right)\\ \; & =\sum_{S\in P\left(I\right)\setminus \emptyset }{\left(-1\right)}^{|S|-1}\int_{0}^{\infty }e^{-\sum_{\xi \in S}\lambda_{\xi }x}dx, \end{array}$$

Solving the integral concludes the proof.

### 7.2. Proof of Theorem 1

To prove the probabilistic bound in Theorem 1, we utilize the well-known *Chebyshev’s inequality*. It provides a handle on the probability that a random variable Z deviates from its mean by more than a given absolute value based on its variance. As given in [55], p. 19, the relation is denoted as follows:

(9) $$\mathrm{Pr}\left(\left|Z-E\left[Z\right]\geq \varepsilon \right|\right)\leq \frac{Var\left[Z\right]}{\varepsilon^{2}}.$$

The probability 1−Pr(j,r) for a certain confidence parameter ϵ represents the likelihood that the upper bound on the time spent in round *r*, i.e., $\mu_{A^{r,★}}\;\;\left(\min\left\{j,T_{r}\right\}-T_{r-1}\right)\;\left(1+\epsilon \right)$, is smaller than the true runtime of the algorithm in iteration *j*, where $j>T_{r-1}$; this can be calculated by applying Chebyshev’s inequality given in (9):

1−Pr(j,r)=Pr(∑y=Tr−1+1minj,TrZAr,★y−μAr,★minj,Tr−Tr−1≥ϵ·μAr,★minj,Tr−Tr−1∑y=Tr−1+1minj,Tr)≤Var∑y=Tr−1+1minj,TrZAr,★yμAr,★2minj,Tr−Tr−12ϵ2≤σAr,★2μAr,★2minj,Tr−Tr−1ϵ2

Then, the probability that $t_{j}$ is underestimated in any of the rounds up to iteration *j* can be given as follows:

$$\begin{array}{cc} 1-\mathrm{Pr}(j) & =1-\prod_{r\in \left[1,b\right]:j>T_{r-1}}\left(1-(1-\mathrm{Pr}(j,r))\right)\\ \; & \leq 1-\prod_{r\in \left[1,b\right]:j>T_{r-1}}\left(1-\frac{\sigma_{A^{r,★}}^{2}}{\mu_{A^{r,★}}^{2}\left(\min\left\{j,T_{r}\right\}-T_{r-1}\right)\epsilon^{2}}\right). \end{array}$$

Note that Pr(j) and Pr(j,r) correspond to the desired events, i.e., the probabilities that the true runtime of the algorithm is less than or equal to the upper bound. Taking the complementary event concludes the proof.

### 7.3. Well-Known Tail Bounds

In Section 7.4, we utilize well-known properties of the tail distributions of sub-gamma and sub-Gaussian random variables, which we provide in the following for completeness.

Sub-gamma tail bound: For $Z∼\mathrm{Sub}\Gamma \left(\alpha ,\beta \right)$ with variance $\sigma^{2}=\frac{\alpha }{\beta^{2}}$ and scale $c=\frac{1}{\beta }$, by [55], p. 29, we have that $\mathrm{Pr}\left(Z>\sqrt{2\sigma^{2}\varepsilon }+c\varepsilon \right)\leq \exp\left(-\varepsilon \right).$

Sub-Gaussian tail bound: Resulting from the Cramér–Chernoff method, we obtain for any $Z∼\mathrm{SubG}\left(\sigma^{2}\right)$ [56], p. 77, that $P\left(Z\leq -\varepsilon \right)\leq \exp\left(-\frac{\varepsilon^{2}}{2\sigma^{2}}\right).$

### 7.4. Proof of Theorem 2

While we will benefit from the proof strategies in [28,38], our analysis differs in that we consider an unbounded distribution of the rewards, which requires us to investigate the properties of sub-gamma distributions and to use different confidence bounds. Also, compared to [28], we deal with LCBs instead of UCBs since we want to minimize the response time of a superarm, i.e., the time spent per iteration. This problem setting was briefly discussed in [38]. While the authors bound the probability of overestimating an entire suboptimal superarm in [38], we bound the probability of individual suboptimal arm choices. This is justified by independent outcomes across arms and by the combined outcome of a superarm being a monotonically non-decreasing function of the individual arms’ rewards, that is, the workers’ mean response times. A superarm $A^{r}\left(j\right)$ is considered suboptimal if $\mu_{A^{r}\left(j\right)}>\mu_{A^{r,★}}$ and a single arm $A_{\nu }^{r}\left(j\right)$ is suboptimal if $\mu_{A_{\nu }^{r}\left(j\right)}>\mu_{A_{\nu }^{r,★}}$. In addition to the counter $T_{i}\left(j\right)$, for every arm i∈[n], we introduce the counter $C_{i,e}\left(j\right)\leq T_{i}\left(j\right)$. The integer *e* refers to the maximum cardinality of all possible superarm choices, i.e., $C_{i,e}\left(j\right)$ is valid for all rounds r≤e. If a suboptimal superarm $A^{r}\left(j\right)$ is chosen, $C_{i,e}\left(j\right)$ is incremented only for the arm $i\in A^{r}\left(j\right)$ that has been pulled the least until this point in time, i.e., $i=A_{\nu_{\min}}^{r}\left(j\right)$, where $\nu_{\min}=\arg\min_{\nu \in A^{r}\left(j\right)}T_{\nu }\left(j\right)$. Hence, $\sum_{i=1}^{n}C_{i,e}\left(j\right)$ equals the number of suboptimal superarm pulls. Note that although there exists an optimal superarm, it is not necessarily unique, i.e., there might exist several superarms $A^{r}\left(j\right)$ with $\mu_{A^{r}\left(j\right)}=\mu_{A^{r,★}}$. Let $W^{\leq e}$ be the set of all superarms with a maximum cardinality of *e*, i.e., $W^{\leq e}≔{⋃}_{r\leq e}W^{r}$, and $T_{A}\left(j\right)$ the number of times superarm A has been pulled until iteration *j*. We have the following:

(10) $$\sum_{U\in W^{\leq e}:\mu_{U}>\mu_{A^{\left|U\right|,★}}}E\left[T_{U}\left(j\right)\right]=\sum_{i=1}^{n}E\left[C_{i,e}\left(j\right)\right].$$

Applying Lemma 4.5 from [56] to express the regret in terms of the suboptimality gaps in iteration *j* of round *r*, i.e., $T_{r-1}<j\leq T_{r}$ and e=r, we use (10) and obtain the following:

(11) $$\begin{array}{cc} R_{j}^{\pi_{\mathrm{cr}}}\; & =\sum_{U\in W^{\leq e}:\mu_{U}>\mu_{A^{\left|U\right|,★}}}\Delta_{U}\;E\left[T_{U}\left(j\right)\right]\\ \; & \leq \max_{r\in \left[1,b\right]:j>T_{r-1}}\Delta_{A^{r},\max}\;\cdot \;\sum_{i=1}^{n}E\left[C_{i,e}\left(j\right)\right]. \end{array}$$

To conclude the proof of Theorem 2, we need the following intermediate results:

**Lemma 1.**  *Let $\left|A^{r}\left(y\right)\right|\leq e$ hold $\forall y\in \left[j\right]$. For any h≥0, we can bound the expectation of $C_{i,e}\left(j\right)$, $i\in \left[n\right]$, as*

$$\begin{array}{cc} E\left[C_{i,e}\left(j\right)\right]\leq \; & \;h+\sum_{y=1}^{j}j^{2}\;\cdot \;\sum_{\nu =1}^{\left|A^{r}\left(y\right)\right|}\mathrm{Pr}\left(\mu_{A_{\nu }^{r}\left(y\right)}>\mu_{A_{\nu }^{r,★}},{\mathrm{LCB}}_{A_{\nu }^{r}\left(y\right)}\left(y\right)\leq {\mathrm{LCB}}_{A_{\nu }^{r,★}}\left(y\right)\right). \end{array}$$

By construction, for a superarm $A^{r}\left(j\right)$ with $\left|A^{r}\left(j\right)\right|\leq e$, it holds for all $\nu \in [1,\left|A^{r}\left(j\right)\right|]$ and $i\in A^{r}\left(j\right)$ that $T_{A_{\nu }^{r}\left(j\right)}\left(j\right)\geq C_{i,e}\left(j\right)$. By applying Lemma 1 with h≥0, we have $T_{A_{\nu }^{r}\left(j\right)}\left(j\right)\geq C_{i,e}\left(j\right)\geq h$. To bound the probability of choosing a suboptimal arm over which we sum in Lemma 1, we use Lemma 2.

**Lemma 2.**  *The probability of the ν-th fastest arm of $A^{r}\left(j\right)$ being suboptimal given that $T_{A_{\nu }^{r}\left(j\right)}\left(j\right)\geq \left\lceil \frac{48\log(j)}{\min\{\delta_{\min}^{2},\delta_{\min}\}}\right\rceil$, is bounded from above as follows:*

(12) $$\begin{array}{cc} \; & \mathrm{Pr}\left(\mu_{A_{\nu }^{r}\left(j\right)}>\mu_{A_{\nu }^{r,★}},{\mathrm{LCB}}_{A_{\nu }^{r}\left(j\right)}\left(j\right)\leq {\mathrm{LCB}}_{A_{\nu }^{r,★}}\left(j\right)\right)\leq j^{-4\min\left\{\lambda_{\min},\lambda_{\min}^{2}\right\}}+j^{-4\lambda_{\min}}. \end{array}$$

Having the results of Lemmas 1 and 2, and choosing $h=\lceil \frac{48\log(j)}{\min\{\delta_{\min}^{2},\delta_{\min}\}}\rceil$, for $\lambda_{\min}\geq 1$, we have the following:

(13) $$\begin{array}{cc} E\left[C_{i,e}\left(j\right)\right] & \leq h+\sum_{y=1}^{j}j^{2}\;\cdot \;\sum_{\nu =1}^{\left|A^{r}\left(y\right)\right|}\mathrm{Pr}\left(\mu_{A_{\nu }^{r}\left(y\right)}>\mu_{A_{\nu }^{r,★}},{\mathrm{LCB}}_{A_{\nu }^{r}\left(y\right)}\left(y\right)\leq {\mathrm{LCB}}_{A_{\nu }^{r,★}}\left(y\right)\right)\\ \; & \leq \left\lceil \frac{48\log(j)}{\min\{\delta_{\min}^{2},\delta_{\min}\}}\right\rceil +\sum_{y=1}^{j}j^{2}\;\cdot \;\sum_{\nu =1}^{e}2j^{-4\lambda_{\min}}\\ \; & \leq \frac{48\log(\min\left\{j,T_{r}\right\})}{\min\left\{\delta_{\min}^{2},\delta_{\min}\right\}}+1+e\;\cdot \;\frac{\pi }{3}, \end{array}$$

where the last step relates to the Basel problem of a *p*-series. We need $\lambda_{\min}\geq 1$ so that the *p*-series converges to a small value. Plugging the bound in (13) into (11) concludes the proof.

The proof of Lemma 1 uses standard techniques from the literature on MABs and is given in the following for completeness. The proof of Lemma 2 is given afterwards.

**Proof of Lemma 1.**  At first, we bound the counter $C_{i,e}\left(j\right)$ from below by introducing an arbitrary parameter *h* that eventually serves to limit the probability of choosing a suboptimal arm. Similar to [38], we have the following:

$$\begin{array}{cc} & C_{i,e}\left(j\right)\leq \begin{array}{cc} & h+\sum_{y=1}^{j}1\left\{\exists \;1\leq \nu \leq \left|A^{r}\left(y\right)\right|:\right.\left.\mu_{A_{\nu }^{r}\left(y\right)}>\mu_{A_{\nu }^{r,★}},{\mathrm{LCB}}_{A_{\nu }^{r}\left(y\right)}\left(y\right)\right.\left.\leq {\mathrm{LCB}}_{A_{\nu }^{r,★}}\left(y\right)\right\} \end{array}\\ & \;\;\;\;\leq \begin{array}{cc} & h+\sum_{y=1}^{j}\sum_{\nu =1}^{\left|A^{r}\left(y\right)\right|}1\left\{\mu_{A_{\nu }^{r}\left(y\right)}>\mu_{A_{\nu }^{r,★}},{\mathrm{LCB}}_{A_{\nu }^{r}\left(y\right)}\left(y\right)\leq {\mathrm{LCB}}_{A_{\nu }^{r,★}}\left(y\right)\right\} \end{array}\\ & \;\;\;\;\leq \begin{array}{cc} & h+\sum_{y=1}^{j}\sum_{\nu =1}^{\left|A^{r}\left(y\right)\right|}1\left\{\mu_{A_{\nu }^{r}\left(y\right)}>\mu_{A_{\nu }^{r,★}},\min_{h\leq T_{A_{\nu }^{r}\left(y\right)}\left(y\right)\leq j}{\mathrm{LCB}}_{A_{\nu }^{r}\left(y\right)}\left(y\right)\leq \max_{1\leq T_{A_{\nu }^{r,★}}\left(y\right)\leq j}{\mathrm{LCB}}_{A_{\nu }^{r,★}}\left(y\right)\right\} \end{array}\\ & \;\;\;\;\leq \begin{array}{cc} & h+\sum_{y=1}^{j}\sum_{T_{A_{\nu }^{r}\left(y\right)}\left(y\right)=h}^{j}\sum_{T_{A_{\nu }^{r,★}}\left(y\right)=1}^{j}\sum_{\nu =1}^{\left|A^{r}\left(y\right)\right|}1\left\{\mu_{A_{\nu }^{r}\left(y\right)}>\mu_{A_{\nu }^{r,★}},{\mathrm{LCB}}_{A_{\nu }^{r}\left(y\right)}\left(y\right)\leq {\mathrm{LCB}}_{A_{\nu }^{r,★}}\left(y\right)\right\} \end{array}\\ & \;\;\;\;\leq \begin{array}{cc} & h+\sum_{y=1}^{j}j^{2}\sum_{\nu =1}^{\left|A^{r}\left(y\right)\right|}1\left\{\mu_{A_{\nu }^{r}\left(y\right)}>\mu_{A_{\nu }^{r,★}},{\mathrm{LCB}}_{A_{\nu }^{r}\left(y\right)}\left(y\right)\leq {\mathrm{LCB}}_{A_{\nu }^{r,★}}\left(y\right)\right\}, \end{array} \end{array}$$

where the first line describes the event of choosing a suboptimal superarm in terms of the events that the LCB of any suboptimal ν-fastest arm in $A^{r}\left(j\right)$ is less than or equal to the LCBs of the ν-fastest arm in $A^{r,★}$. To conclude the proof, we take the expectation on both sides. □

**Proof of Lemma 2.**  As given in [28], to overestimate the ν-th fastest arm of $A^{r}\left(j\right)$—i.e., for ${\mathrm{LCB}}_{A_{\nu }^{r}\left(j\right)}\left(j\right)\leq {\mathrm{LCB}}_{A_{\nu }^{r,★}}\left(j\right)$ to hold—at least one of the following events must be satisfied:

(14) $$\begin{array}{cc} \mu_{A_{\nu }^{r,★}} & >\mu_{A_{\nu }^{r}\left(j\right)}-2\theta_{A_{\nu }^{r}\left(j\right)}\left(j\right), \end{array}$$

(15) $$\begin{array}{cc} {\hat{\mu }}_{A_{\nu }^{r,★}}\left(j\right) & \geq \mu_{A_{\nu }^{r,★}}+\theta_{A_{\nu }^{r,★}}\left(j\right), \end{array}$$

(16) $$\begin{array}{cc} {\hat{\mu }}_{A_{\nu }^{r}\left(j\right)}\left(j\right) & \leq \mu_{A_{\nu }^{r}\left(j\right)}-\theta_{A_{\nu }^{r}\left(j\right)}\left(j\right). \end{array}$$

In the following, let f(j)≔2log(j). We first show that the requirement—namely, $T_{A_{\nu }^{r}\left(j\right)}\left(j\right)\geq \left\lceil \frac{24f(j)}{\min\{\delta_{\min}^{2},\delta_{\min}\}}\right\rceil ≔\ell$—guarantees that $\mu_{A_{\nu }^{r,★}}-\mu_{A_{\nu }^{r}\left(j\right)}+2\theta_{A_{\nu }^{r}\left(j\right)}\left(j\right)\leq 0$, for all $A^{r}\left(j\right)\in W^{r}$ with r∈[1,b], ν∈[1,r] and $\mu_{A_{\nu }^{r}\left(j\right)}>\mu_{A_{\nu }^{r,★}}$, thus making the event (14) a zero-probability event. The scaling factor γ=24 is chosen as an approximation of the exact solution of $2\left(\sqrt{\frac{4}{\gamma }}+\frac{2}{\gamma }\right)=1$, which is $4\left(3+2\sqrt{2}\right)\approx 23.31\leq 24$. We have the following:

$$\begin{array}{cc} \mu_{A_{\nu }^{r,★}} & -\mu_{A_{\nu }^{r}\left(j\right)}+2\left(\sqrt{\frac{4f(j)}{T_{A_{\nu }^{r}\left(j\right)}\left(j\right)}}+\frac{2f(j)}{T_{A_{\nu }^{r}\left(j\right)}\left(j\right)}\right)\\ \; & \leq \mu_{A_{\nu }^{r,★}}-\mu_{A_{\nu }^{r}\left(j\right)}+2\left(\sqrt{\frac{4f(j)}{h}}+\frac{2f(j)}{h}\right)\\ \; & \leq -\delta_{A_{\nu }^{r}\left(j\right)}+\delta_{\min}\leq 0. \end{array}$$

**Lemma 3.**  *Given i.i.d. random variables $Z_{i}^{j}∼\exp\left(\lambda_{i}\right)$, j=1,…,T, the deviation of the empirical mean from the true mean ${\hat{\mu }}_{i}-\mu_{i}≔\frac{1}{T}\sum_{j=1}^{T}\left(Z_{i}^{j}-E\left[Z_{i}^{j}\right]\right)$ follows a sub-gamma distribution $\mathrm{Sub}\Gamma \left(T,T\lambda_{i}\right)$ on the right tail and a sub-Gaussian distribution $\mathrm{SubG}\left(\frac{1}{T\lambda_{i}^{2}}\right)$ on the left tail.*

We apply Lemma 3 (which is proven below) to bound the probability of the event (15). However, two cases have to be distinguished. For $\lambda_{\min}\geq 1$, we have the following:

$$\begin{array}{cc} \; & \mathrm{Pr}\left({\hat{\mu }}_{A_{\nu }^{r,★}}\left(j\right)\geq \mu_{A_{\nu }^{r,★}}+\theta_{A_{\nu }^{r,★}}\left(j\right)\right)\\ \; & =\mathrm{Pr}\left({\hat{\mu }}_{A_{\nu }^{r,★}}\left(j\right)\geq \mu_{A_{\nu }^{r,★}}+\sqrt{\frac{4f(j)}{T_{A_{\nu }^{r,★}}\left(j\right)}}+\frac{2f(j)}{T_{A_{\nu }^{r,★}}\left(j\right)}\right)\\ \; & \leq \mathrm{Pr}\left({\hat{\mu }}_{A_{\nu }^{r,★}}\left(j\right)\geq \mu_{A_{\nu }^{r,★}}+\sqrt{\frac{4f(j)}{T_{A_{\nu }^{r,★}}\left(j\right)\lambda_{\min}}}+\frac{2f(j)}{T_{A_{\nu }^{r,★}}\left(j\right)}\right)\\ \; & \leq \mathrm{Pr}\left({\hat{\mu }}_{A_{\nu }^{r,★}}\left(j\right)\geq \mu_{A_{\nu }^{r,★}}\;+\;\sqrt{\frac{4f\left(j\right)\lambda_{\min}}{T_{A_{\nu }^{r,★}}\left(j\right)\;\lambda_{\min}^{2}}}\;+\;\frac{2f\left(j\right)\lambda_{\min}}{T_{A_{\nu }^{r,★}}\left(j\right)\;\lambda_{\min}}\right)\\ \; & =\mathrm{Pr}\left({\hat{\mu }}_{A_{\nu }^{r,★}}\left(j\right)\geq \mu_{A_{\nu }^{r,★}}\;+\;\sqrt{\frac{8\log\left(j\right)\lambda_{\min}^{2}}{T_{A_{\nu }^{r,★}}\left(j\right)\lambda_{\min}^{2}}}+\frac{4\log\left(j\right)\lambda_{\min}^{2}}{T_{A_{\nu }^{r,★}}\left(j\right)\lambda_{\min}}\right)\\ \; & \leq \exp-4\log\left(j\right)\lambda_{\min}\leq j^{-4\lambda_{\min}}, \end{array}$$

where—at the penultimate step—we apply the sub-gamma tail bound given in Section 7.3 with $\varepsilon =2\log\left(j\right)\lambda_{\min}$.

In contrast, if $\lambda_{\min}<1$

$$\begin{array}{cc} \; & \mathrm{Pr}\left({\hat{\mu }}_{A_{\nu }^{r,★}}\left(j\right)\geq \mu_{A_{\nu }^{r,★}}+\theta_{A_{\nu }^{r,★}}\left(j\right)\right)\\ \; & =\mathrm{Pr}\left({\hat{\mu }}_{A_{\nu }^{r,★}}\left(j\right)\geq \mu_{A_{\nu }^{r,★}}+\sqrt{\frac{4f(j)}{T_{A_{\nu }^{r,★}}\left(j\right)}}+\frac{2f(j)}{T_{A_{\nu }^{r,★}}\left(j\right)}\right)\\ \; & \leq \mathrm{Pr}\left({\hat{\mu }}_{A_{\nu }^{r,★}}\left(j\right)\geq \mu_{A_{\nu }^{r,★}}+\sqrt{\frac{4f(j)}{T_{A_{\nu }^{r,★}}\left(j\right)}}+\frac{2f\left(j\right)\lambda_{\min}}{T_{A_{\nu }^{r,★}}\left(j\right)}\right)\\ \; & =\mathrm{Pr}\left({\hat{\mu }}_{A_{\nu }^{r,★}}\left(j\right)\geq \mu_{A_{\nu }^{r,★}}+\sqrt{\frac{4f\left(j\right)\lambda_{\min}^{2}}{T_{A_{\nu }^{r,★}}\left(j\right)\lambda_{\min}^{2}}}+\frac{2f\left(j\right)\lambda_{\min}^{2}}{T_{A_{\nu }^{r,★}}\left(j\right)\lambda_{\min}}\right)\\ \; & =\mathrm{Pr}\left({\hat{\mu }}_{A_{\nu }^{r,★}}\left(j\right)\geq \mu_{A_{\nu }^{r,★}}+\sqrt{\frac{8\log\left(j\right)\lambda_{\min}^{2}}{T_{A_{\nu }^{r,★}}\left(j\right)\lambda_{\min}^{2}}}+\frac{4\log\left(j\right)\lambda_{\min}^{2}}{T_{A_{\nu }^{r,★}}\left(j\right)\lambda_{\min}}\right)\\ \; & \leq \exp-4\log\left(j\right)\lambda_{\min}^{2}\\ \; & \leq j^{-4\lambda_{\min}^{2}}, \end{array}$$

where—at the penultimate line—we use the sub-gamma tail bound in Section 7.3 with $\varepsilon =2\log\left(j\right)\lambda_{\min}^{2}$.

For the event in (16), we have the following:

$$\begin{array}{cc} \; & \mathrm{Pr}\left({\hat{\mu }}_{A_{\nu }^{r}\left(j\right)}\left(j\right)\leq \mu_{A_{\nu }^{r}\left(j\right)}-\theta_{A_{\nu }^{r}\left(j\right)}\left(j\right)\right)\\ \; & =\mathrm{Pr}\left({\hat{\mu }}_{A_{\nu }^{r}\left(j\right)}\left(j\right)\leq \mu_{A_{\nu }^{r}\left(j\right)}-\sqrt{\frac{4f(j)}{T_{A_{\nu }^{r}\left(j\right)}\left(j\right)}}-\frac{2f(j)}{T_{A_{\nu }^{r}\left(j\right)}\left(j\right)}\right)\\ \; & \leq \mathrm{Pr}\left({\hat{\mu }}_{A_{\nu }^{r}\left(j\right)}\left(j\right)\leq \mu_{A_{\nu }^{r}\left(j\right)}-\sqrt{\frac{4f(j)}{T_{A_{\nu }^{r}\left(j\right)}\left(j\right)}}\right)\\ \; & \leq \exp-4\lambda_{A_{\nu }^{r}\left(j\right)}^{2}\log\left(j\right)\\ \; & \leq j^{-4\lambda_{A_{\nu }^{r}\left(j\right)}^{2}}\leq j^{-4\lambda_{\min}^{2}}\leq j^{-4\lambda_{\min}}, \end{array}$$

where we use the sub-Gaussian tail bound given in Section 7.3. □

**Proof of Lemma 3.**  To conduct the proof, we express the independently and identically distributed random variables $Z_{i}^{j}∼\exp\left(\lambda_{i}\right)$, j∈[1,T] in terms of its moment generating functions $M_{Z_{i}}\left(\phi \right)=E\left[e^{\phi Z_{i}}\right]=\frac{\lambda_{i}}{\lambda_{i}-\phi }$, which is defined for ϕ<λ. Summing the identically distributed realizations is equivalent to multiplying their moment generating functions, i.e., $\sum_{j=1}^{T}Z_{i}^{j}↔\prod_{j=1}^{T}M_{Z_{i}^{j}}\left(\phi \right)={\left(\frac{\lambda_{i}}{\lambda_{i}-\phi }\right)}^{T}$, which describes a random variable related to a gamma distribution with shape *T* and rate $\lambda_{i}$. The empirical mean ${\hat{\mu }}_{i}=\frac{1}{T}\sum_{j=1}^{T}Z_{i}^{j}$ is, thus, distributed according to the scaled gamma distribution $\Gamma (T,\lambda_{i}T)$ with mean $\mu_{i}=\frac{1}{\lambda_{i}}$ and variance $\sigma^{2}=\frac{1}{T\lambda_{i}^{2}}$. Thus, ${\hat{\mu }}_{i}-\mu_{i}$ is a centered gamma distributed random variable, which, according to [55], p. 27, has the properties stated in Lemma 3. □

### 7.5. Proof of Theorem 3

In the following, we prove the regret bounds given in Theorem 3 based on the analysis in [29]. In particular, we adapt the regret proof in [29] for single-arm choices to prove a combinatorial regret bound. To start with, we use the same counter definitions as in Section 7.4, and follow the same steps—i.e., we apply Lemma 4.5 from [56] and use the interdependencies between the counters—to finally arrive at (17). Let $e=\max_{r\in \left[1,b\right]:j>T_{r-1}}r$, then we have the following:

(17) $$\begin{array}{cc} R_{j}^{\pi }\; & \leq \max_{r\in \left[1,b\right]:j>T_{r-1}}\Delta_{A^{r},\max}\;\cdot \;\sum_{i=1}^{n}E\left[C_{i,e}\left(j\right)\right]. \end{array}$$

To conclude, we need to bound the expectation of $C_{i,e}\left(j\right)$. A handle on this is given in the following Lemma 4.

**Lemma 4.**  *The expectation of the suboptimal arm counter $C_{i,e}\left(j\right)$ for superarms $A^{r}\left(y\right)$, $y\in \left[j\right]$, with maximum cardinality e, i.e., $\forall y\in \left[1,j\right]$, $\left|A^{r}\left(r\right)\right|\leq e$, LCBs according to (7), and $f\left(j\right)=\log\left(j\right)+3\log\left(\log\left(j\right)\right)$, j>3, can be bounded as follows:*

$$\begin{array}{cc} E\left[C_{i,e}\left(j\right)\right] & \leq e\left(7\log\left(\log\left(j\right)\right)+\frac{1+\epsilon }{D_{\mathrm{KL},\min}}f\left(j\right)+Q_{j}\left(\epsilon \right)\right), \end{array}$$

*where $Q_{j}\left(\epsilon \right)=\frac{\exp\left(-D_{\mathrm{KL}\epsilon ,\min}\left(\frac{1+\epsilon }{D_{\mathrm{KL},\max}}f\left(j\right)-1\right)\right)}{1-\exp(-D_{\mathrm{KL}\epsilon ,\min})}$.*

Plugging the result of Lemma 4 into (17) concludes the proof of Theorem 3. It remains to prove Lemma 4.

**Proof of Lemma 4.**  To prove Lemma 4, we need a result from [29], which we state in our notation and for our problem in Lemma 5. We briefly explain why this result holds in our setting.

**Lemma 5**  ([29])**.** *The probability of overestimating the ν-th fastest element of $A^{r}\left(j\right)$ in any of the iterations up to iteration j, given that the element is suboptimal, under a decision process $\pi_{\mathrm{kl}}$ based on the LCB in (7) and $K_{j}=\left\lfloor \frac{1+\epsilon }{D_{\mathrm{KL}}^{-}\left(\mu_{A_{\nu }^{r}\left(j\right)}∥\mu_{A_{\nu }^{r,★}}\right)}f\left(j\right)\right\rfloor$ can be upper-bounded as follows:*

$$\begin{array}{cc} \sum_{y=1}^{j} & \mathrm{Pr}\left(\mu_{A_{\nu }^{r}\left(y\right)}>\mu_{A_{\nu }^{r,★}},{\mathrm{LCB}}_{A_{\nu }^{r}\left(y\right)}\left(y\right)\leq {\mathrm{LCB}}_{A_{\nu }^{r,★}}\left(y\right)\right)\\ \; & \leq 7\log\left(\log\left(j\right)\right)+\frac{1+\epsilon }{D_{\mathrm{KL}}^{-}\left(\mu_{A_{\nu }^{r}\left(j\right)}∥\mu_{A_{\nu }^{r,★}}\right)}f\left(j\right)+\frac{\exp\left(-D_{\mathrm{KL}}\left(\phi \left(\epsilon ,\mu_{A_{\nu }^{r,★}},\mu_{U_{\nu }}\right)∥\mu_{A_{\nu }^{r}\left(j\right)}\right)K_{j}\right)}{1-\exp(-D_{\mathrm{KL}}\left(\phi \left(\epsilon ,\mu_{A_{\nu }^{r,★}},\mu_{U_{\nu }}\right)∥\mu_{A_{\nu }^{r}\left(j\right)}\right)}, \end{array}$$

*where $\phi (\epsilon ,\mu_{A_{\nu }^{r,★}},\mu_{A_{\nu }^{r}\left(j\right)})$, defined on the open interval $]\mu_{A_{\nu }^{r,★}},\mu_{A_{\nu }^{r}\left(j\right)}[$, is calculated such that $D_{\mathrm{KL}}\left(\phi \left(\epsilon ,\mu_{A_{\nu }^{r,★}},\mu_{A_{\nu }^{r}\left(j\right)}\right)∥\mu_{A_{\nu }^{r,★}}\right)=D_{\mathrm{KL}}\left(\mu_{A_{\nu }^{r}\left(j\right)}∥\mu_{A_{\nu }^{r,★}}\right)/\left(1+\epsilon \right)$.*

**Sketch of Proof.**  The authors of [29] bound the probability of choosing a suboptimal arm based on a KL-divergence-based UCB for all distributions being part of the exponential family. As we can mirror the probability density function of the exponential distribution at the y-axis and result in a distribution from the exponential family, we can transfer our LCB setting to an equivalent UCB setting. Thus, by symmetry, the performance guarantees of the non-combinatorial KL-based policy for the exponential family apply. □

Starting with a similar approach as in the proof of Lemma 1 and with the help of the relation given in Lemma 5, we can bound the expectation of the counter $C_{i,e}\left(j\right)$ as follows:

$$\begin{array}{cc} \; & E\left[C_{i,e}\left(j\right)\right]\leq \sum_{y=1}^{j}\mathrm{Pr}\left(\exists \;1\leq \nu \leq \left|A^{r}\left(y\right)\right|:\mu_{A_{\nu }^{r}\left(y\right)}>\mu_{A_{\nu }^{r,★}},{\mathrm{LCB}}_{A_{\nu }^{r}\left(y\right)}\left(y\right)\leq {\mathrm{LCB}}_{A_{\nu }^{r,★}}\left(y\right)\right)\; \end{array}$$

(18) $$\begin{array}{cc} \; & \leq \sum_{y=1}^{j}\sum_{\nu =1}^{\left|A^{r}\left(y\right)\right|}\mathrm{Pr}\left({\mathrm{LCB}}_{A_{\nu }^{r}\left(y\right)}\left(y\right)\leq {\mathrm{LCB}}_{A_{\nu }^{r,★}}\left(y\right),\mu_{A_{\nu }^{r}\left(y\right)}>\mu_{A_{\nu }^{r,★}}\right) \end{array}$$

(19) $$\begin{array}{cc} \; & \leq \sum_{\nu =1}^{e}\sum_{y=1}^{j}\mathrm{Pr}\left({\mathrm{LCB}}_{A_{\nu }^{r}\left(y\right)}\left(y\right)\leq {\mathrm{LCB}}_{A_{\nu }^{r,★}}\left(y\right),\mu_{A_{\nu }^{r}\left(y\right)}>\mu_{A_{\nu }^{r,★}}\right)\\ \; & \leq \sum_{\nu =1}^{e}7\log\left(\log\left(j\right)\right)+\frac{1+\epsilon }{D_{\mathrm{KL}}^{-}\left(\mu_{A_{\nu }^{r}\left(j\right)}∥\mu_{A_{\nu }^{r,★}}\right)}f\left(j\right)+\frac{\exp\left(-D_{\mathrm{KL}}\left(\phi \left(\epsilon ,\mu_{A_{\nu }^{r,★}},\mu_{U_{\nu }}\right)∥\mu_{A_{\nu }^{r}\left(j\right)}\right)K_{j}\right)}{1-\exp(-D_{\mathrm{KL}}\left(\phi \left(\epsilon ,\mu_{A_{\nu }^{r,★}},\mu_{U_{\nu }}\right)∥\mu_{A_{\nu }^{r}\left(j\right)}\right)}\\ \; & \leq e\left(7\log\left(\log\left(j\right)\right)+\frac{1+\epsilon }{D_{\mathrm{KL},\min}}f\left(j\right)+Q_{j}\left(\epsilon \right)\right), \end{array}$$

where from (18) to (19), we use the convention that $1\{{\mathrm{LCB}}_{A_{\nu }^{r}\left(y\right)}\left(y\right)\leq {\mathrm{LCB}}_{A_{\nu }^{r,★}}\left(y\right),\mu_{A_{\nu }^{r}\left(y\right)}>\mu_{A_{\nu }^{r,★}}\}=0$ if $\nu >\left|A^{r}\left(y\right)\right|$. This concludes the proof. □

## 8. Conclusions

In this paper, we introduced a cost-efficient distributed machine learning scheme that assigns random tasks to fast workers and leverages all computations. The number of workers employed per iteration increases as the algorithm evolves. To speed up the convergence, we introduced the use of a CMAB model, for which we provided theoretical regret guarantees and simulation results. While our scheme is inferior to the adaptive *k*-sync strategy from [8] in terms of speed, it achieves a much lower error rate with the same computational efforts while reducing the communication load significantly.

As further research directions, one could derive tighter regret bounds and improve the choice of the confidence bound. In addition, the setting in which the main node assigns a number of computational tasks proportional to the expected speed of the workers could be analyzed. We expect the tools developed in this work to be useful for this more challenging scenario. One can also consider the setting in which the underlying distributions of the response times of the workers vary over time. Furthermore, relaxing the shared memory assumption and instead fixing the task distribution to the workers introduces an interesting trade-off between the average waiting time per iteration and the convergence rate in distributed machine learning with CMABs. At a high level, this holds because the main node should sample different subsets of the data at every iteration. Hence, the main node cannot always employ the fastest workers.

## Data Availability

No new data were created or analyzed in this study. Data sharing is not applicable to this article.

## Abbreviations

The following abbreviations are used in this manuscript:

SGD	stochastic gradient descent
MAB	multi-armed bandit
UCB	upper confidence bound
LCB	lower confidence bound
KL	Kullback–Leibler
CMAB	combinatorial multi-armed bandit
